# Enhancement of carer skills and patient function in the non-pharmacological management of frontotemporal dementia (FTD): A call for randomised controlled studies

**DOI:** 10.1590/S1980-57642013DN70200002

**Published:** 2013

**Authors:** Claire M. O'Connor, Lindy Clemson, Thaís Bento Lima da Silva, Olivier Piguet, John R. Hodges, Eneida Mioshi

**Affiliations:** 1 Ageing, Work & Health Research Unit, Faculty of Health Sciences, University of Sydney.; 2 Neuroscience Research Australia, Sydney, Australia.; 3 Behavioural and Cognitive Neurology Unit, Neurology Department, University of São Paulo, São Paulo SP, Brazil.; 4 School of Medical Science, University of New South Wales, Sydney, Australia.

**Keywords:** frontotemporal dementia, non-pharmacological intervention, functional disability, randomised controlled trial

## Abstract

FTD is a unique condition which manifests with a range of behavioural symptoms, marked dysfunction in activities of daily living (ADL) and increased levels of carer burden as compared to carers of other dementias. No efficacious pharmacological interventions to treat FTD currently exist, and research on pharmacological symptom management is variable. The few studies on non-pharmacological interventions in FTD focus on either the carer or the patients' symptoms, and lack methodological rigour. This paper reviews and discusses current studies utilising non-pharmacological approaches, exposing the clear need for more rigorous methodologies to be applied in this field. Finally, a successful randomised controlled trial helped reduce behaviours of concern in dementia, and through implementing participation in tailored activities, the FTD-specific Tailored Activities Program (TAP) is presented. Crucially, this protocol has scope to target both the person with FTD and their carer. This paper highlights that studies in this area would help to elucidate the potential for using activities to reduce characteristic behaviours in FTD, improving quality of life and the caregiving experience in FTD.

## Abstract (pt)

A DFT é uma condição única que se manifesta por uma variedade de sintomas, principalmente em atividades da vida diária (AVD) e aumento da carga sobre os cuidadores em comparação aos cuidadores de outras demências. Não existe nenhuma intervenção farmacológica para tratamento da DFT até o momento e pesquisas sobre o manejo farmacológico dos sintomas são variáveis. Os poucos estudos em intervenção não farmacológica em DFT focam nos cuidadores ou em sintomas dos pacientes, faltando rigor metodológico. Este artigo revisa e discute os estudos atuais que utilizam abordagem não farmacológica, o que expõe a clara necessidade para metodologias mais rigorosas a serem aplicadas neste campo. Finalmente, um ensaio clinico randomizado bem sucedido ajudou na redução de comportamentos em demência, através da implementação da participação em atividades ajustadas, é apresentado o FTD-specific Tailored Activities Program (TAP). Este protocolo visa abordar tanto o paciente com DFT quanto seu cuidador. Este manuscrito evidencia que pesquisas dentro desta area ajudariam a elucidar o potencial em usar estas atividades para redução dos comportamentos característicos em DFt, melhorando a qualidade de vida e experiências dos cuidadores em DFT.

## INTRODUCTION

rontotemporal dementia (FTD) is a term used to describe a progressive neurodegenerative disorder associated with atrophy in the frontal and temporal lobes of the brain.^1^ The three main clinical variants of FTD are classified based on their early symptoms, comprising a behavioural variant (bvFTD) and two language variants.^2^ The language variants are further classified depending on their pattern of language impairment: semantic dementia (svPPA) and progressive non-fluent aphasia (nfv-PPA).^3,4^ SvPPA is also associated with marked behavioural symptoms.^5,6^ Symptom overlap can occur between the variants as disease spreads later in the course of disease progression.^7,8^ Regardless of variant, FTD affects functional ability from an early stage, especially more complex activities.

In 2011, a set of revised diagnostic criteria was proposed for the bvFTD. With the revised criteria, a diagnosis of "possible" bvFTD requires three of the six clinically discriminated characteristics: loss of inhibition, apathy/inertia, loss of empathy, perseveration/compulsive behaviours, hyperorality and dysexecutive neuropsychological profile. "Probable" bvFTD requires the additional features of functional disability and characteristic neuroimaging, whereas bvFTD "with definitive frontotemporal lobar degeneration" requires histopathological confirmation or evidence of pathogenic mutation.^2,9,10^ Therefore, investigating functionality is essential for the diagnosis and it is also relevant for the treatment of the syndrome, given that the impact on activities of daily living (ADLs) can be used as a clinical parameter.

Functional ability, as measured by ADLs, is more impaired in FTD than in Alzheimer's disease (AD).^11-13^ In a previous study, we found that impairments in complex, instrumental ADLs (IADLs) such as managing finances and medications, are similarly impaired across FTD subtypes, while deficits in basic ADLs (BADLs) such as showering, dressing and eating differ.^12^ Patients with nfv-PPA were found to present with the least change in BADL performance, followed by svPPA, with bvFTD patients being the most impaired subgroup. Over time, however, svPPA have been shown to decline at a slower rate than both bvFTD and nfv-PPA.^14,15^

Impairments in function lead to progressive and marked dependence on the carer; in addition, behavioural symptoms associated with FTD frequently make dementia caregiving even more challenging.^16^ Behavioural symptoms are at the core of FTD and the severity of behavioural symptoms appears to be related to the setting in which the patient lives. People with FTD living at home present with more severe levels of behavioural disturbance than those living in care facilities;^17-19^ however, this is likely to be related to disease staging or use of medication. A study by Mourik et al.^18^ found that carers of people with FTD living at home were more distressed than carers of people living in residential care. This finding, however, is not universal, as demonstrated by a study in England where carers of people with FTD living at home and of those living in residential care shared similar levels of stress and depression.^17^ Finally, carers of people with FTD have been shown to demonstrate higher levels of depression, stress and burden than carers of other types of dementia such as AD.^17,20,21^ Given the severe nature of both behavioural changes and functional deficits, it is not surprising that disease severity, as measured by a combination of behaviour and functional impairments, should be the main contributor to burden in FTD carers, rather than just one of these aspects alone.^22^

The unique devastating impact that FTD has on both the person with FTD and their carers demonstrates the great need for interventions targeted to this specific dementia syndrome. Pharmacological interventions in FTD have yet to be shown to provide any clear benefit,^23^ which may be related to the highly heterogeneous nature of FTD pathology.^24^ This lack of effective treatment in FTD has been exemplified by a recent multi-centre, randomised, double-blind, placebo-controlled trial of the drug memantine.^25^ Results from this study found the intervention group to have a greater decline in mental speed and more frequent adverse cognitive side effects than the placebo group. Pharmacological interventions currently used in FTD are therefore primarily targeted at treating specific behavioural symptoms such as agitation, aggression or obsessive behaviour.^26,27^ Large scale, randomised, double-blind, placebo-controlled trials to prove the effectiveness of these pharmacological drugs have yet to be conducted.^23,28^ Investigations into several medications have had mixed results in FTD including some showing adverse side effects such as worsening of symptoms.^28^

It is clear that the development of appropriate non-pharmacological interventions in FTD is of the utmost importance. This paper will argue for the importance of non-pharmacological interventions to improve patient function in FTD - through the integration of addressing both patient behaviour and carer issues, while reviewing studies which have been centred on carer issues or patient behaviour to date.

## METHODS

A search of MEDLINE, PubMed, Cochrane database, and CINAHL was conducted up until 30^th^ January 2013. The search terms frontotemporal dementia, frontotemporal lobar degeneration, and Pick's disease, were combined with the terms treatment, intervention, non-pharmacological, support, carer, caregiver, and therapy. Articles were considered for inclusion if they discussed non-pharmacological interventions within an FTD cohort. Due to the lack of studies with rigorous methodology for clinical trials, studies of all levels of evidence (n=16) which met these inclusion criteria were considered for review. In other words, the number of papers available was so small that we included them all in this review. It should be noted that two of these studies considered for review were written in Japanese and thus were not accessed in full text.

**Non-pharmacological interventions in FTD.** It is becoming increasingly recognised that people with FTD and their carers have unique needs for support. These include skills to manage profound behavioural and language changes, issues surrounding finances and availability of appropriate care services as a result of the often younger onset of the disease, combined with greater levels of carer stress and burden than in other types of dementia such as AD.^29-32^ Perhaps not surprisingly, FTD carers appear significantly less satisfied with support from specialist health care providers than AD carers.^33^ This finding highlights the lack of appropriate services that address specific issues in FTD, and the pressing need for further investigation into FTD-specific non-pharmacological intervention strategies. Mendez^34^ reported that non-pharmacological management strategies in FTD should primarily include behavioural interventions, education and a focus on the carer. To date, crucially, no randomised controlled trials have taken place to evaluate the benefit of any non-pharmacological interventions in FTD. Table 1 summarises all current studies available.

**Table 1:** Summary of current studies of non-pharmacological interventions for FTD.

Author Date	Intervention	Target	Methodology	Level Evidence (NHMRC)	Sample size	Outcomes
Litvan2001	• Potential use of neurotransmitter replacement, biologic treatment, and carer management	• Approaches to improve FTD management	• Expert opinion; no data	**	N/A	• N/A
Lough & Hodges2002	• Behavioural modification techniques	• Behavioural management	• Case report	IV	1	• Specific BPSD were altered with the patient through use of behavioural modification techniques
Diehl et al. 2003a2003b	•Support group for FTD carers (providing information/advice/support) • Structured to provide educational and therapeutic sessions • 7 weekly sessions	• Carer education	• Pilot; Mixed methods study with one treatment group; Qualitative	IV	8	• Improved understanding and knowledge of FTD • Less than half learnt strategies to care for themselves
Banks et al. 2006	•3-part series of FTD-specific conferences for carers over one year • One hour lecture followed by health care worker-run support group	• Carer education and support	• Descriptive; Qualitative	**	• Session 1 (n=55) • Session 2 (n=51) • Session 3 (n=48)	• Some carers reported information and support was positive, while some gave negative feedback regarding impracticality of session content
Merrilees2007	• Potential use of A-B-C Model	• Behavioural management	• Expert opinion; no data	**	N/A	• N/A
Grinberg et al.2008 andGrinberg & Phillips2009	• Integrating a day program specialised for persons with FTD into an already established day program • Tailor-made individual, small group and specialised activities • Staff strategies to generate increased self-worth and accomplishment • Education/support offered to family/carers • Behaviour management techniques and supportive/enabling approach to address needs of FTD patients and their families'	• Behavioural management • Carer education and respite • Increase QOL for patients, family and carers	• Descriptive; Qualitative	**	6	• Qualitatively reported to be positive outcomes • Issues with frequent predominance of older group members and distance to attend
Yamakawa et al. 2008	• Environmental intervention A (doors shut to remove stimulus of seeing beds to sleep on during the day) and intervention B (staff walking with participant to increase activity levels) were compared	• Behavioural management	• Objective (power IC tags) and subjective measures; SSD	IV	1	• Sleep-wake cycles restored with intervention A; with intervention B nighttime ambulation increased significantly
• Marziali and Climans2009	• Online video conferencing education and support group for FTD spousal carers	• Carer education and support	• Feasibility study; qualitative	**	6	• Carers reported social support and accessibility as positive aspects of the intervention • Reduced burden was reported • Levels of stress remained unchanged
Chow et al.2011	• Web-based anonymous survey developed specifically for FTD carers to investigate need for FTD carer support resources	• Guide for available resources	• Cross sectional online survey	IV	78*	• Family counselling and public education regarding FTD identified as priorities by carers
Raglio et al. 2012	•Active Music therapy (interaction with music therapist using instruments and vocals) •50 therapy sessions over 6 months	• Behavioural management	• Case report	IV	1	• Reduced global scores for NPI, CMAI and CSDD (>50% reduction)
Mioshi et al. 2012	• Structured FTD carer group program (problem solving/re-framing/seeking support) • 15 weekly sessions	• Carer education, coping and management strategies	• Pilot; comparative study	III-2	21	• Intervention group carers demonstrated reduced burden (ZBI) and reaction to behaviours (CBI-R); maintained at 12 months. No change reported on DASS Change in humour on COPE
McKinnon et al. 2013	• Structured FTD carer group program (cognitive appraisal/coping strategies) • 15 weekly sessions	• Carer education, coping and management strategies	• Pilot; Qualitative; comparative study	III-2	21	• Intervention group carers improved by 63% in functional responses on fictitious scenario compared with only 13% of those in control group

Levels of evidence based on the National Health and Medical Research Council (NHMRC) evidence hierarchy (66): I=systematic review, II=randomised controlled trial, III 1=pseudorandomised controlled trial, III-2=comparative study with concurrent controls, III 3=comparative study without concurrent controls, IV=case series with either post-test or pre test/post-test outcomes.

** This study does not fulfil any of the criteria recommended by the NHMRC evidence hierarchy.

* Only 62 completed entire survey. FTD: frontotemporal dementia; BPSD: behavioural and psychological symptoms of dementia; SSD: single subject design; QOL: quality of life; NPI: Neuropsychiatric Inventory; CMAI: Cohen-Mansfield Agitation Inventory; CSDD: Cornell Scale for Depression in Dementia; ZBI: Zarit Burden Inventory; CBI-R: Cambridge Behavioural Inventory Revised; DASS: Depression, Anxiety and Stress Scale; COPE: Cope questionnaire.

**Carer-based interventions.** *Interventions promoting education.* German studies^35,36^ have reported on an FTD-specific carer support group (n=8) aimed at providing information, advice and support. Seven physician-facilitated structured sessions were conducted, which were designed to be both educational and therapeutic. Outcomes were analysed immediately post intervention via interviews, and again after six months via mailed questionnaires, with a qualitative approach. Overall, carers reported improved understanding and knowledge of the disease, but less than half reported having learnt strategies to care for themselves better as carers. Nonetheless, no control group was included in the study.

With similar aims to the German studies, Banks et al.^29^ reported on an intervention based on a series of FTD-specific conferences for FTD carers, which incorporated an educational lecture session followed by a facilitated support group. Evaluation forms were used to gather qualitative feedback from carers who attended these sessions. While some feedback was positive regarding information and support provided at the sessions, other feedback was rather negative and stated that the lecture content was impractical. This study also did not have a control group, and lacked quantification from which to obtain greater interpretation of the results.

An online video-conferencing education and support group for FTD spousal carers (n=6), which involved 10 weekly one-hour sessions facilitated by a health-care professional was trialled in Canada.^37^ This online intervention was based on evidence from efficacious dementia carer support groups previously reported,^38,39^ where these groups were adapted to suit FTD cohorts. Qualitative analysis conducted post intervention (via interview) identified that carers reported social support and accessibility as positive aspects to the intervention. Reduced burden was also reported; levels of stress, however, remained unchanged, and the study lacked a control group to avoid the Hawthorne effect, where bias may have been introduced by participants knowingly receiving an intervention.^40^

*Structured interventions promoting carers' skills.* Riedijk et al.^19^ reported that carers who use passive coping strategies were more likely to have high levels of burden and decreased health-related quality of life (QOL). The importance of carer education and identifying support services was also highlighted by Mohandras and Rajmohan.^41^ Following on from these, a recent study investigated the impact of a structured intervention program for FTD carers to specifically teach skills on cognitive appraisal and coping strategies, including education on seeking support. The main outcomes were reduction of burden and enhancement of coping skills.^42^ The intervention was based on a previously developed intervention for general dementia carers,^43^ and adapted to suit FTD populations. The program involved an intervention group (n=9) and a control group (n=12), and was run in weekly sessions for 15 weeks (2 hours each). The study reported that both carer burden and carer reaction to behaviours decreased significantly in the intervention group, which persisted at 12 months follow-up.^42^ Transferability of the skills learnt in the intervention was demonstrated through the qualitative analysis of a fictitious problem-solving scenario.^44^ However, similarly to the qualitatively reported results from Marziali and Climans,^37^ no change in carer stress was identified, and the participants were similarly not randomised to intervention or control groups.

Despite these positive outcomes generated from carer-based interventions, the studies mentioned above are of a limited transferability due to limitations in study design, such as lack of randomisation, and small sample sizes. Rigorous studies are therefore required to reproduce the promising findings, and to further support the establishment of carer-based interventions in FTD.

**Patient-based interventions.** The current lack of randomised controlled trials on non-pharmacological interventions in FTD extends to patient-based interventions. In this area, the majority of studies have been based on either case studies or narratives and expert opinion articles.

Behavioural modification interventions have been described, where a multidisciplinary FTD-specific day-program was implemented within an existing day program at an adult day centre.^30,45^ The aim of the program was very broad, offering individualised activities to patients, specific FTD-related training for staff members, as well as provision of education and support to family carers. Preliminary outcomes of the program are reported to be positive. The authors also discussed frequent limitations of such programs for people with FTD due to the frequent predominance of older group members, or to distance.^30^ This important variable highlights the need for development of community-based (local) FTD specific interventions to facilitate accessibility to carers.

Yamakawa et al.^46^ reported on a single-subject design study where an environmental intervention was used with an FTD patient to restore sleep-wake cycles. The intervention of shutting doors during the day was shown to significantly reduce the patient's evening and night-time disturbances. Lough and Hodges^47^ also published a case report describing strategies for behavioural and psychological symptoms of dementia (BPSD) management in a patient with FTD. In the study, behaviour modification techniques were found to be effective for altering the expression of specific BPSD exhibited by the participant; quantitative outcome measures to support these findings were, however, missing. A recent case study^48^ reported on the use of active music therapy to reduce BPSD in a patient with FTD. The result with this case was the reduction of BPSD by more than 50%. Taken together, these single-subject design studies suggest that interventions focused on patient behaviours may be beneficial in FTD. Future studies should focus on rigorous methodology.

## Expert advice on non-pharmacological interventions to date.

Litvan^49^ highlighted the need for improved carer management in FTD, recommending carer education, emotional support, treatment of psychiatric morbidities, and provision of respite care. The need to offer appropriate support for FTD carers was also discussed by a Canadian exploratory study suggesting the internet as a platform for accessible FTD educational resources.^50^

The antecedent-behaviour-consequences (ABC) model is an approach used by family carers for management of behavioural symptoms in FTD.^32^ In this model, brain atrophy is considered the antecedent [A]; behavioural symptoms secondary to this atrophy is considered the behaviour [B], and carer response to this behaviour is the consequence [C]. Specific examples of BPSD were used to explicate this behavioural modification based intervention, with suggested interventions ranging from environmental, behavioural, pharmacologic and physical.^51^ Although these approaches seem coherent in the FTD context, a pressing need for research studies to verify the efficacy of these recommendations exists.

**Improving function in FTD: an intervention that integrates patient and carer issues concomitantly.** To date, most interventions in FTD have focused on either carer burden or behavioural disturbances, with limited number of studies with interventions focused on activity. Two Japanese studies aimed at promoting improved function have been found in FTD. Ikeda et al.^52^ reported on the benefit of individualised activities based on previous interests for reducing BPSD in people with FTD (n=4), while another study presented an OT approach^53^ involving family education in conjunction with patient-based interventions to increase QOL and improve care in FTD. However, limited information is available as both studies were published in Japanese journals.

A promising intervention, the Tailored Activities Program (TAP), has yet to be trialled in FTD. This approach incorporates the aforementioned concepts of using individualised activities with patients in conjunction with carer education, and thus shows great potential for success in this cohort. Developed by Gitlin et al.,^54^ TAP is a community-based occupational therapy based intervention designed to reduce BPSD by prescribing personalised activities. Importantly, these activities are based on preserved capabilities and previous interests and roles, with scope for transferability as the dementia progresses.

Crucially, the TAP pilot study^54^ was a two-group randomised controlled trial (n=60) conducted with general dementia patients. Results demonstrated a treatment effect for reduced incidences of BPSD overall, with significant decreases in shadowing, repetitive questioning, agitation and argumentation. Following the intervention, carers reported greater activity engagement and ability for patients to keep busy, as well as fewer hours on duty or doing things for the patient. Carer skills increased through increased mastery, self-efficacy using activities, and greater use of simplification techniques. These initial outcomes of enhanced activity engagement and reduced BPSD along with enhanced caregiving skills, suggest TAP warrants consideration as a good candidate for non-pharmacological intervention within FTD populations living in the community. FTD-specific services, as well as general services able to cater for FTD patients are lacking.^55^ Development of an FTD-specific TAP protocol potentially provides translational opportunity for the development of an appropriate community-based intervention for people with FTD.

TAP requires active carer involvement, from activity development to generalisation of strategies and downgrading of activity for future decline in function. Similar use of problem solving strategies seem to have contributed to the positive carer outcomes reported by Mioshi et al.,^42^ and Robinson.^56^ Both studies have highlighted the importance of FTD carers to learn problem solving techniques to develop strategies to assist with patient function by themselves. This finding appears to be the key to success, because carers are instilled with an increased sense of control by taking an active approach to resolving specific problems. This concept was also suggested by Talerico and Evans,^57^ who proposed working with carers to implement personalised environmental and behavioural interventions to promote improved function and QOL in a person with FTD.

One of the challenges to improve function in FTD is to address the marked frontal deficits that characterise the disease, such as apathy and executive dysfunction.^58,59^ The TAP intervention involves structuring activities based on single rather than multiple tasks,^60^ a strategy also suggested by Massimo and Grossman^27^ to assist with impairments in executive functioning in FTD.^2,59^ Other studies^61^ have also advocated the importance of providing interventions which match the functional level of the person with FTD, while Robinson^56^ proposed the potential use of strategies to enhance attention and use procedural/implicit learning strategies despite the degenerative nature of FTD. Moreover, the TAP intervention also involves setting up the environment to facilitate initiation and sequencing,^60^ another potentially important consideration in FTD where apathy frequently prevents initiation of tasks,^58,62^ and executive dysfunction and utilisation behaviour may impede the continuation of an activity.^27,63^

The paucity of reliable, accessible information regarding services for FTD dyads living in the community is an issue discussed by a number of studies.^33,35,50,57^ These studies have argued that carers need detailed information about the condition in order to improve their understanding of the disease process, and thus better cope with their own emotional reactions. This concept leads to the recommendation to involve professionals with expert knowledge in FTD to work with affected families for greater efficacy.^61^ The TAP intervention involves providing general dementia information to carers, which can therefore be tailored for FTD-specific information.

Finally, TAP also includes carer stress-reducing techniques, an important consideration in FTD interventions,^21,42,61^ especially in light of marked levels of stress reported by carers.^64,65^

**Conclusions and future directions.** FTD is a unique condition which manifests with a range of behavioural symptoms,^59^ marked ADL dysfunction^12^ and increased levels of carer burden as compared to carers of other dementias such as AD.^22^ No efficacious pharmacological interventions to treat FTD exist, and research involving non-pharmacological interventions to manage symptoms is variable. A few studies regarding non-pharmacological interventions in FTD have been published; however, the need for rigorous methodologies to be applied is clear. A promising approach is the use of an FTD-specific TAP protocol, which can target both the person with FTD and their carer. Such a program has scope to elucidate the potential for using activity to improve patient function and reduce behavioural changes, while improving carers' skills and reducing difficulties in the caregiving experience in FTD.
